# The Potential Target Value of ADP-Ribosylation Factor 6 in Insulin Secretion Regulation and the Treatment of Metabolic Disorders

**DOI:** 10.3390/metabo16070508

**Published:** 2026-07-21

**Authors:** Yangyang Wang

**Affiliations:** Jilin Collaborative Innovation Center for Antibody Engineering, Jilin Medical University, Jilin 132013, China; wyystudent@163.com

**Keywords:** ADP-ribosylation factor 6, insulin secretion, metabolic disorders, type 2 diabetes mellitus, obesity

## Abstract

Obesity and type 2 diabetes mellitus (T2DM) represent pandemic metabolic illnesses hallmarked by defective pancreatic β-cell function and blunted insulin release. As a conserved small GTPase (guanosine triphosphatase), ADP-ribosylation factor 6 (ARF6) governs fundamental cellular events encompassing vesicle trafficking, cytoskeleton remodeling and lipid metabolic turnover. Emerging data confirm that ARF6 acts as a master rheostat of glucose-stimulated insulin secretion (GSIS) in β-cells through downstream cell division control protein 42/Ras-related C3 botulinum toxin substrate 1 (Cdc42/Rac1) cascades. Pathogenic ARF6 hyperactivation triggers a cascade of β-cell lesions: mitochondrial impairment, autophagic suppression and exacerbated inflammatory signaling, accelerating the progression of obesity and T2DM. First-line therapeutics ranging from GLP-1 (Glucagon-like peptide-1) receptor agonists and metformin to SGLT2 (Sodium-Glucose Cotransporter 2) inhibitors partially restore metabolic homeostasis by rectifying aberrant ARF6-dependent signaling axes. This review comprehensively delineates ARF6’s canonical cellular roles, mechanistic bridges connecting ARF6 to β-cell failure and metabolic deterioration, and functional crosstalk between ARF6 and established anti-metabolic pharmacotherapies. We further address unresolved research gaps and prospective translational avenues, offering actionable perspectives to advance ARF6 as a tractable therapeutic target for obesity and T2DM management.

## 1. Introduction

ADP-ribosylation factor 6 (ARF6) is a central member of the small GTPase family [1], with key functions including involvement in vesicle trafficking, cytoskeletal rearrangement, and lipid metabolism regulation [2]. Recent studies have highlighted the critical role of ARF6 in metabolic regulation, particularly in the GSIS process in pancreatic β-cells [3]. ARF6 regulates vesicle fusion and insulin granule release through interactions with Cdc42 and Rac1 [4,5,6], thereby exerting a profound impact on metabolic homeostasis. However, metabolic disorders, such as obesity and T2DM, have become leading public health issues worldwide, contributing significantly to mortality and disability. The core pathological features of T2DM include insulin resistance (IR) and dysfunction of pancreatic β-cells [7,8], which exacerbate the risk of chronic hyperglycemia and increase the incidence of cardiovascular diseases, non-alcoholic fatty liver disease (NAFLD), and neuropathy [9,10,11,12]. Research demonstrates a strong association between T2DM and obesity, with obesity promoting the development of IR through ectopic fat deposition and chronic inflammation [13,14]. Certain genetic variants, such as GCKR rs1260326, are considered to play a significant role in NAFLD and T2DM associated with obesity [15]. These findings highlight the necessity for further exploration of the underlying molecular mechanisms [16].

Furthermore, mitochondrial dysfunction plays a pivotal role in the pathophysiology of T2DM and obesity. Studies show that overexpression of ferritin in adipose tissue can induce mitochondrial dysfunction [17] and, through compensatory mechanisms, significantly increase the number of pancreatic β-cells. This phenomenon suggests that ARF6 may play an important role in the signaling axis between adipose tissue and the pancreas, thereby regulating metabolic homeostasis under metabolic stress conditions [18]. Additionally, mitochondrial calcium signaling plays a crucial role in regulating insulin secretion. Disruption of calcium homeostasis damages mitochondrial health and further impairs β-cell function, with ARF6 potentially influencing insulin secretion and metabolic homeostasis via calcium-dependent signaling mechanisms [19].

**Figure 1:**
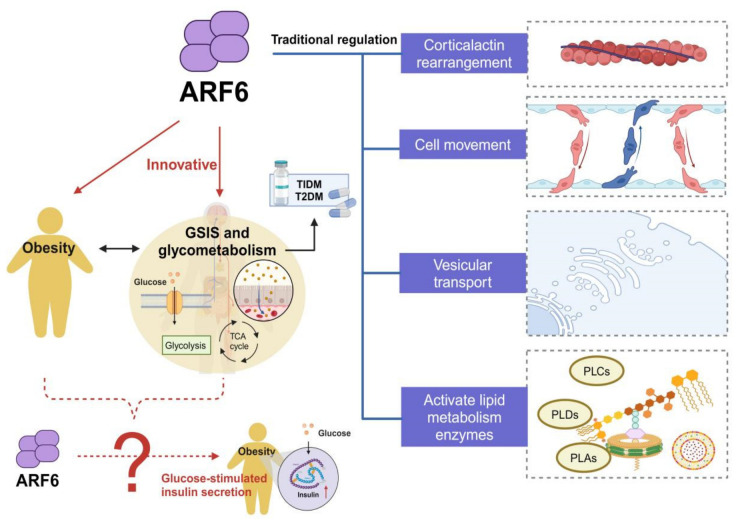
**Schematic overview of ARF6 biological functions.** Blue branches represent its well-established canonical activities, including cortical actin rearrangement, cell movement, vesicular transport, and activation of lipid metabolic enzymes [phospholipase C (PLCs), PLDs, PLAs]. The red innovative axis illustrates a newly identified pathogenic cascade: ARF6 modulates GSIS and glycometabolism in pancreatic β-cells, mediating the bidirectional interplay between obesity and the progression of T2DM. A potential direct regulatory axis from ARF6 to obesity-impaired insulin secretion is also proposed (marked with dashed lines).

Earlier reviews analyze ARF6 functions separately. They either discuss insulin secretion or adipose metabolism, but never treat ARF6 as a core link between obesity and T2DM [20] (Figure 1). These reviews also have two clear limitations. First, they do not rank anti-diabetic drugs by the strength of evidence linking them to ARF6. Second, they rarely mention ARF6’s tissue-specific dual functions and U-shaped activity curve. Both gaps slow the clinical translation of ARF6-targeted therapies. This review resolves these deficits by establishing an integrated cascade connecting ARF6 overactivation to β-cell impairment, adipose lipotoxicity, mitochondrial dysfunction and systemic inflammation to recapitulate obesity-T2DM progression; stratifying ten categories of anti-metabolic agents by the robustness of ARF6-associated experimental evidence to discriminate direct biochemical regulation from indirect pathway correlation; and unpacking translational limitations stemming from tissue-dependent opposing ARF6 functions and its narrow physiological activity range while proposing multi-omics, human organoid and tissue-targeted drug development strategies to advance ARF6 toward clinical therapeutic application.

## 2. Overview

Distinct from autoimmune type 1 diabetes (T1DM) with absolute insulin loss, T2DM develops from combined IR and gradual β-cell secretory exhaustion. Lifestyle intervention and hypoglycemic drugs can partially reverse early metabolic dysfunction, while advanced stages feature irreversible β-cell dedifferentiation and cell death.

### 2.1. ARF6 and T2DM

ARF6 plays a key role in activating phospholipase D1 and phospholipid synthesis, which is critical for maintaining plasma membrane integrity [21]. Additionally, ARF6 modulates various cellular processes, including autophagy [22] and tumor invasion and migration [23], through interactions with downstream effectors such as Cdc42 and Rac1. Studies also show that ARF6 regulates the transport of palmitoylated proteins from the Golgi apparatus to the plasma membrane, a function with significant implications for signal transduction and molecular-targeted therapy [24,25]. Upon activation by inflammatory signals, ARF6 significantly increases vascular permeability and endothelial cell leakage, accelerating the onset of vascular diseases and dysfunction [26,27]. Dysregulation of ARF6 function is closely associated with the development of various diseases, including tumor metastasis, neurodegenerative disorders, and metabolic disorders [21]. Additionally, ARF6 activation promotes cancer cell proliferation and survival by modulating the Rac1 signaling pathway, suggesting it may be an important therapeutic target for developing novel protein–protein interaction inhibitors [28].

In pathological models of diabetes, ARF6 dysfunction can lead to disrupted insulin secretion and decreased survival of pancreatic β-cells. Mouse models with ARF6 knockout exhibit significant defects in insulin secretion, further confirming the central role of ARF6 in metabolic homeostasis [29]. Moreover, ARF6 dysfunction has been linked to the dedifferentiation of pancreatic β-cells and ferroptosis, mechanisms that are considered crucial in the development of T2DM. These findings closely associate ARF6 with T2DM, as its function directly affects pancreatic β-cell activity and insulin secretion regulation. Further investigation into the specific mechanisms by which ARF6 contributes to metabolic disorders is crucial for understanding the pathophysiology of T2DM and developing new intervention strategies. In recent years, novel antidiabetic drugs, such as SGLT2 inhibitors, have shown significant efficacy in improving diabetic complications. These medications reduce hyperglycemia and decrease fluid overload, substantially improving diabetes-related cardiovascular complications [30]. Additionally, studies highlight the importance of integrating lifestyle interventions, including healthy eating and regular physical activity, with pharmacological treatments for managing the long-term prognosis of T2DM [21].

Although research on the role of ARF6 in T2DM is limited, existing evidence suggests that it plays a crucial role in regulating pancreatic β-cell function. ARF6 influences β-cell cytoskeletal remodeling and insulin granule release by modulating the Cdc42 and Rac1 signaling pathways [29]. Additionally, ARF6 dysfunction in pancreatic β-cells may be closely linked to ferroptosis associated with obesity, contributing to insulin secretion dysfunction in T2DM [31]. Recent studies further indicate that abnormalities in the ARF6 signaling pathway may exacerbate β-cell dysfunction by affecting lipid metabolism and inflammatory signaling pathways [24]. These findings underscore the potential importance of ARF6 in the pathogenesis of T2DM.

The core characteristics of T2DM include IR and pancreatic β-cell dysfunction. While IR has long been considered the primary pathophysiological feature of T2DM, recent research highlights the critical role of functional β-cell defects in disease progression. In particular, the loss of insulin secretion capacity is one of its hallmark features [32]. β-cell dedifferentiation, apoptosis, and chronic stress induced by glucotoxicity and lipotoxicity further exacerbate this dysfunction [33]. Weight gain and the accumulation of pancreatic fat significantly contribute to β-cell dysfunction. Ectopic fat deposition increases oxidative stress and inflammatory signaling, amplifying β-cell damage. This pathological mechanism may be linked to the abnormal expression of genes related to insulin secretion [32]. In diabetic models, β-cell dedifferentiation is associated with disrupted glycolysis and mitochondrial function, leading to a marked reduction in GSIS. In T2DM patients, reducing hyperglycemia significantly improves β-cell function and delays disease progression. Studies show that dietary interventions, such as low-carbohydrate diets and regular exercise, can improve insulin sensitivity and restore β-cell metabolic function [34]. Additionally, novel drugs such as GLP-1RA and SGLT2 inhibitors have demonstrated significant efficacy in promoting insulin secretion and improving β-cell function [35]. Research also indicates that T2DM patients experience a decline in β-cell compensatory capacity, which impairs their ability to respond fully to the metabolic stress caused by IR [33]. Therefore, targeting key nodes in the GSIS pathway and enhancing mitochondrial activity may represent important future directions for T2DM therapy.

**Figure 2:**
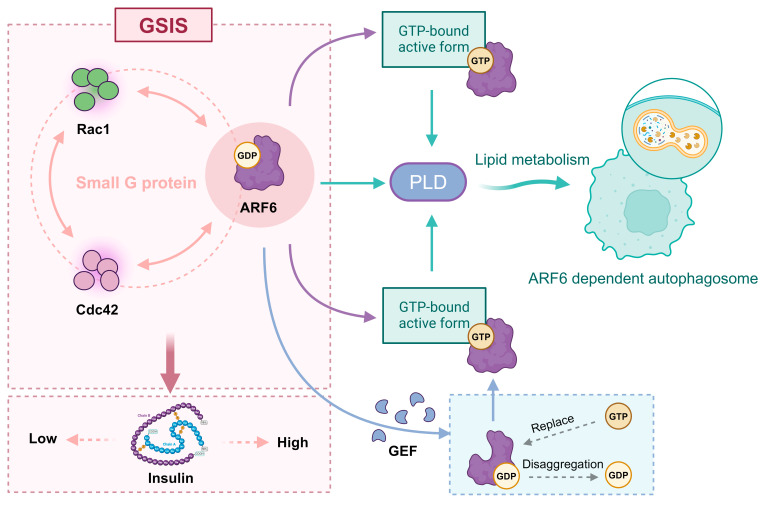
**Relationship between ARF6 and glucose metabolism.** The small GTPases ARF6, Cdc42, and Rac1 mediate the potential interaction model for GSIS. Glucose metabolism activates Cdc42 and ARF6, activating lipid metabolism enzymes in β-cells. ARF6 cycles between its active form, bound to guanosine triphosphate (GTP), and its inactive form, bound to guanosine diphosphate (GDP). Conformational changes in ARF6 facilitate autophagy in a GTP-dependent manner. Additionally, ARF6 regulates membrane flow by affecting the activity of PLD and modulating autophagy. ARF6 also promotes GDP dissociation and GTP exchange by regulating guanine nucleotide exchange factor (GEFs).

The small G protein ARF6 plays a crucial role in regulating GSIS. ARF6 promotes insulin secretion by activating the Cdc42 and Rac1 signaling pathways, thereby regulating vesicle fusion and cytoskeletal remodeling in β cells [36,37] (Figure 2). Studies demonstrate that a significant reduction in GSIS is one of the hallmark features of T2DM, and ARF6 dysfunction may exacerbate β-cell dysfunction by interfering with lipid metabolism and mitochondrial activity [32].

Recent studies have further elucidated the role of ARF6 in β-cell dedifferentiation and cellular stress. For example, under chronic hyperglycemic conditions, aberrant activation of ARF6 may impair cellular metabolic homeostasis through the Rac1-mediated oxidative stress signaling pathway [38]. Additionally, ARF6 has been implicated in ferroptosis, a process considered a key mechanism underlying β-cell dysfunction in T2DM. Although research on the relationship between ARF6 and insulin secretion has been increasing, studies directly investigating the specific role of ARF6 in T2DM remain limited. Future in-depth analysis of the ARF6 signaling pathway will provide valuable insights into its potential as a therapeutic target.

### 2.2. ARF6 and Obesity

**Figure 3:**
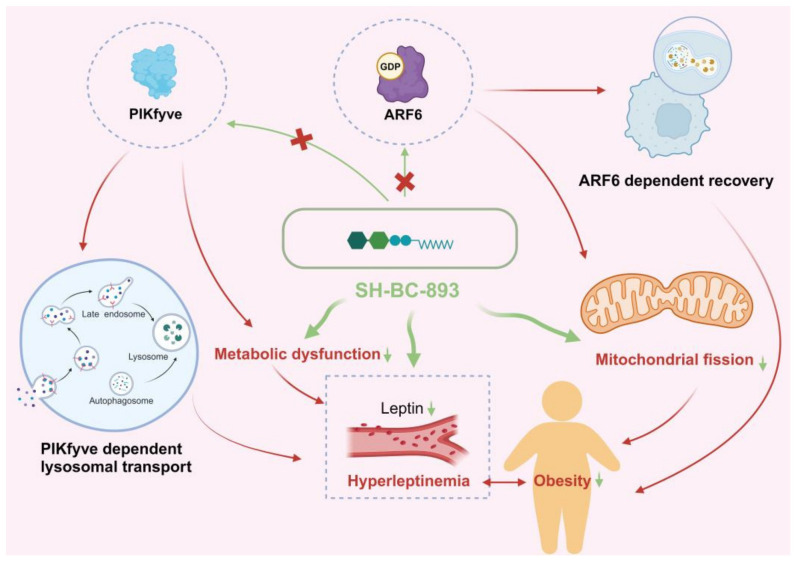
**The mechanism by which SH-BC-893 blocks mitochondrial fission through modulation of related factors.** SH-BC-893 (C19H32ClNO) reverses obesity and metabolic dysfunction in mice by blocking mitochondrial fission. Its mechanism includes the reversal of hyperlipidemia and mitochondrial fragmentation in the hypothalamus and the rapid inhibition of ceramide-induced mitochondrial fission through the suppression of ARF6-dependent recycling processes and PIKfyve-dependent lysosomal trafficking. Red crosses denote the inhibitory actions of SH-BC-893: the upper cross represents inhibition of the ARF6-dependent recycling process, and the left cross represents inhibition of PIKfyve-dependent lysosomal trafficking.

ARF6 is closely associated with T2DM, a condition that often coexists with obesity, one of the primary risk factors for T2DM. This suggests that ARF6 may play a crucial role in obesity-related metabolic disorders. One study demonstrated that the ceramide analog SH-BC-893 effectively inactivates ARF6, thereby blocking mitochondrial fission and disrupting the endocytic cycle, which significantly reduces food intake [39]. This effect also protects mitochondrial function and improves metabolic homeostasis by inhibiting ARF6-dependent recycling processes. SH-BC-893 enhances metabolic function by blocking the phosphoinositide kinase FYVE domain-containing protein (PIKfyve)-dependent lysosomal fusion reaction [40]. In a high-fat diet (HFD) mouse model, SH-BC-893 reversed obesity and associated metabolic dysfunction by inhibiting ARF6- and PIKfyve-dependent transport events [41]. The compound improved liver and brain mitochondrial morphology by preventing mitochondrial fission induced by palmitic acid and ceramides and restored mitochondrial function in white adipose tissue. Additionally, SH-BC-893 corrected abnormal plasma leptin and adiponectin levels in obese mice, significantly restoring glucose metabolism and liver lipid homeostasis within 4 h of oral administration [40]. Recent studies further underscore the key role of ARF6 in regulating metabolic pathways and organelle function. For example, ARF6 modulates dynamic changes in cell membrane lipids via its endocytic and recycling pathways, influencing lipid metabolism and insulin sensitivity [42]. Additionally, SH-BC-893’s protective effect on mitochondrial function provides further evidence of its potential in treating obesity-related diseases (Figure 3). These findings not only highlight ARF6 as a potential target for regulating obesity and metabolic function but also lay the foundation for developing novel intervention strategies, such as ceramide analogs. However, further research is needed to fully elucidate the specific role of ARF6 in the molecular mechanisms underlying obesity.

ARF6 plays a critical role in GSIS in pancreatic β-cells, although its precise mechanisms remain incompletely understood [43]. ARF6 has been identified as a key regulator of GSIS, modulating insulin granule release through the activation of the Cdc42 and Rac1 signaling pathways [44,45]. The ARF6 (T27N) mutation significantly reduces GSIS without affecting the half-maximal effective concentration of glucose, suggesting that its regulatory role is primarily focused on granule transport and release stages [5]. ARF6 activity cycles between GDP-bound inactive and GTP-bound active states, modulated by ARNO (GEF) and ARF GAPs (GTPase-activating protein) [5]; lipid modifications control its membrane shuttling. Activated ARF6 upregulates PLD to enrich PI(4,5)P2 lipid microdomains [46]. It sequentially activates Cdc42 and Rac1: Cdc42 drives local actin polymerization, while Rac1 rearranges the cytoskeletons to mobilize insulin granules [47]. Finally, ARF6 coordinates Rab27a and SNARE complexes to facilitate granule–plasma membrane fusion for GSIS [48].

An organism’s metabolic and energy status largely depends on the availability of substrates, such as glucose, to generate ATP for maintaining physiological functions. Imbalances in glucose homeostasis can lead to pathological conditions, including cardiovascular failure and metabolic diseases such as diabetes and obesity [49]. Currently, some traditional medications used to treat T2DM may induce side effects with long-term use, such as the risk of hypoglycemia, weight gain, and renal dysfunction [50]. Therefore, developing novel therapeutic agents has become critical for T2DM management. Moreover, the future of metabolic disease treatment may include personalized therapies based on precision medicine. By integrating patients’ genetic backgrounds, metabolic states, and lifestyle factors, emerging gene-editing technologies and peptide-based drugs are being explored to optimize treatment options further [51].

In fact, since the 1970s, significant progress has been made in developing glucose-sensitive insulin (GSI) formulations. The core mechanism of these formulations is to encapsulate insulin in polymers, enabling glucose-dependent dynamic release [52]. Analyses reveal that these innovative formulations release insulin in response to blood glucose spikes through polymer-responsive modules, thus avoiding the risk of hypoglycemia associated with traditional insulin therapy [53]. The success of GSI offers a precise and dynamic solution for diabetes treatment. In recent years, metformin, exenatide (a GLP-1 analog), and MK-626 (a dipeptidyl peptidase 4 inhibitor) have been shown to promote autophagy in pancreatic β-cells and enhance insulin secretion. As a crucial cellular mechanism, autophagy maintains β-cell homeostasis and balance by clearing damaged organelles and proteins. These studies highlight the indispensable role of autophagy in insulin regulation and metabolic health [42]. Exercise increases whole-body metabolic efficiency through insulin-independent glucose uptake and significantly improves insulin sensitivity and glucose clearance [54]. Additionally, exercise activates autophagy pathways, enhancing β-cell resistance to oxidative stress and improving insulin secretion function [55]. These findings further emphasize the importance of exercise as a non-pharmacological intervention in diabetes management.

Although research on the role of ARF6 in β-cell metabolic regulation remains limited, evidence suggests that it participates in the regulation of insulin secretion and autophagy pathways through interactions with GEFs [42]. Early evidence indicates that abnormal activation of ARF6 may interfere with the membrane transport system in β-cells, thereby reducing the efficiency of GSIS [29]. Additionally, ARF6 activation is linked to lipid metabolism and oxidative stress-related signaling pathways, providing scientific rationale for its potential as a target for diabetes therapy.

## 3. Potential Link Between ARF6 and Obesity and T2DM Medications

We classified anti-obesity and anti-diabetic agents into three tiers based on experimental evidence linking their efficacy to ARF6 signaling to distinguish direct physical regulation, mandatory indirect mediation, and non-causal parallel pathway overlap:

Tier 1 (Direct biochemical validation): Compounds with confirmed physical interaction with ARF6/ARNO GEF and quantifiable modulation of ARF6 GTP-GDP cycling via co-immunoprecipitation and GTP pull-down assays.

Tier 2 (Indirect essential mediator): Drugs whose therapeutic effects are fully blunted under ARF6 knockout, confirming ARF6 as an obligate downstream signaling intermediate.

Tier 3 (Correlative parallel pathways only): Agents showing synchronized ARF6 pathway fluctuation without biochemical or genetic proof of causal ARF6 dependence.

**Figure 4:**
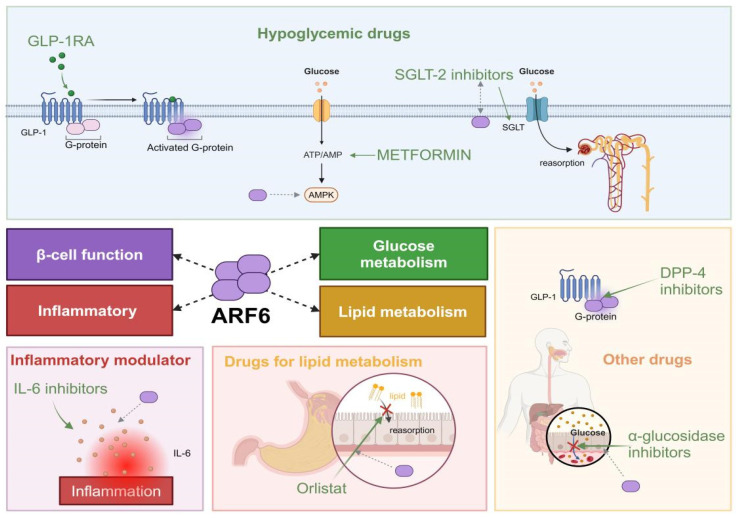
**Association between ARF6, obesity, and T2DM treatments.** Schematic illustrating the core regulatory axes of ARF6 (β-cell function, inflammation, and glucose and lipid metabolism) and corresponding antidiabetic pharmacotherapies. Representative agents include GLP-1RAs, metformin, SGLT2 inhibitors, DPP-4 inhibitors, α-glucosidase inhibitors, orlistat, and IL-6 inhibitors, which act via distinct molecular and physiological pathways to counteract ARF6-mediated diabetic pathogenic processes. Dashed lines = regulatory/interaction pathways; solid lines = drug action pathways.

Several known medications to treat T2DM and obesity may be directly or indirectly related to ARF6, with mechanisms that could be further elucidated. However, these associations may only manifest as indirect effects in certain individual drugs. For example, the following commonly used drugs may involve ARF6 (Figure 4):

(1) Liraglutide (Tier 1, Direct validated modulator): Liraglutide is a GLP-1 receptor agonist that lowers blood glucose and reduces body weight by improving systemic insulin sensitivity [56]. Biochemical co-immunoprecipitation assays confirm physical complex formation between GLP-1R and the ARF6 guanine nucleotide exchange factor ARNO, which blocks ARNO-mediated GDP-GTP exchange on ARF6 [5]. GTP pull-down experiments further quantify the reduction of active GTP-bound ARF6 after liraglutide exposure, providing direct biochemical proof that liraglutide modulates ARF6 nucleotide cycling. Endothelial-specific ARF6 overexpression drives systemic insulin resistance in chow-fed mice, a phenotype fully rescued by liraglutide treatment, confirming ARF6 as a direct functional effector of GLP-1RA metabolic benefits [20].
(2) Metformin (Tier 2, Indirect essential mediator): Metformin inhibits hepatic glucose production and enhances peripheral insulin sensitivity via the AMP-activated protein kinase (AMPK) pathway [57]. Activated AMPK fine-tunes physiological ARF6 GTP-GDP recycling to sustain intact glucose-stimulated insulin secretion (GS) [42]. In ARF6-knockout pancreatic β-cells and mouse islets, metformin fails to restore impaired GSIS, demonstrating ARF6 is an obligate downstream signaling intermediate rather than a mere parallel signaling readout [5].
(3) Alpha-glucosidase inhibitors (Tier 3, Correlative parallel pathways only): Alpha-glucosidase inhibitors delay intestinal carbohydrate digestion to suppress postprandial hyperglycemia [58]. Although ARF6 and Rac1 jointly control β-cell GSIS machinery, no co-immunoprecipitation, ARF6 activity pull-down, or ARF6 knockout functional assays have validated causal regulation between this drug class and ARF6. Observed simultaneous shifts in glucose metabolism and ARF signaling only reflect overlapping metabolic networks without directional causal linkage [59].

(4) Bile acid sequestrants (Tier 3, Correlative parallel pathways only): Bile acid sequestrants improve glucose homeostasis by remodeling intestinal bile acid signaling [60]. ARF6 preserves gut barrier integrity through Sirt1-dependent cascades to support insulin sensitivity, yet no biochemical binding tests or ARF6 loss-of-function studies confirm that bile acid sequestrants target ARF6 as a core therapeutic mechanism [20]. The shared metabolic outputs arise from independent parallel signaling axes rather than ARF6-dependent drug action.
(5) Dopamine agonists (Tier 3, Correlative parallel pathways only): Dopamine agonists mitigate insulin resistance and restrain hepatic glucose overproduction. The ARF6 effector ASAP1 participates in dopaminergic metabolic circuits, but no genetic ablation or protein interaction evidence verifies ARF6 as a required intermediate to mediate the hypoglycemic effects of dopamine agonists [61]. Current literature only documents non-causal pathway correlation.
(6) SGLT-2 inhibitors (Tier 2, Indirect essential mediator): SGLT-2 inhibitors reduce glycemia by inhibiting renal tubular glucose reabsorption [52]. ARF6 subcellular membrane localization governs renal epithelial glucose transport function. Global or renal tubular-specific ARF6 knockout blunts both the glucose-lowering and reno-protective effects of SGLT-2 inhibitors in preclinical models, verifying ARF6 acts as an indispensable downstream mediator of this drug class [62].
(7) Orlistat (Tier 3, Correlative parallel pathways only): Orlistat blocks gastrointestinal lipase activity to reduce dietary fat absorption [63]. While ARF6 controls membrane lipid remodeling, there exists no direct biochemical evidence for physical binding between orlistat and ARF6 or its GEF regulators [64]. Concurrent alterations in lipid turnover and ARF6 signaling after treatment represent coincident pathway covariation, not ARF6-dependent therapeutic machinery.
(8) GLP-2 analogs (Tier 3, Correlative parallel pathways only): GLP-2 analogs improve metabolic profiles by reinforcing intestinal epithelial barrier function [65]. Few published co-immunoprecipitation or ARF6 knockout functional research confirms GLP-2 signaling directly reshapes ARF6 nucleotide cycling. Any regulatory connection between GLP-2 and ARF6 remains an unsubstantiated hypothesis lacking experimental causal proof.
(9) IL-6 inhibitors (Tier 3, Correlative parallel pathways only): IL-6 inhibitors restore insulin sensitivity by suppressing chronic low-grade inflammation [66]. ARF6 drives proinflammatory NF-κB cascades, yet no ARF6 ablation studies demonstrate that IL-6 blockade relies on ARF6 suppression to reverse insulin resistance [67]. Only synchronized fluctuations in inflammatory signaling have been recorded, without validated causal ARF6 mediation [20].
(10) Inhibitors of small G proteins (Tier 1, Direct validated modulator): Selective ARF6 antagonists directly bind ARF6 or its GEF proteins to lock ARF6 in the inactive GDP-bound conformation [68]. In vitro co-immunoprecipitation and GTP pull-down assays confirm direct physical binding and robust suppression of ARF6 GTP loading. In high-fat-diet obese mouse models, these compounds reverse adipose and β-cell metabolic dysfunction by inhibiting pathological ARF6 hyperactivation, with direct ARF6 inhibitory activity fully biochemically validated.

Collectively, Tier 1 agents possess definite biochemical evidence for ARF6 nucleotide cycle regulation; Tier 2 drugs rely on ARF6 as a non-negotiable downstream effector to exert metabolic benefits; Tier 3 compounds merely share overlapping metabolic signaling networks without causal linkage to ARF6 activity. This tiered classification eliminates ambiguous wording such as “potential link” or “indirect involvement” and clearly discriminates causal ARF6 mediation from coincident signaling covariation.

## 4. Discussion

In summary, ARF6 governs pancreatic β-cell function via a U-shaped activity-response gradient [28]. Subphysiological, GDP-locked ARF6 suppresses Cdc42/Rac1-dependent vesicle transport and autophagy, impairing GSIS; balanced physiological GTP-GDP ARF6 cycling sustains steady insulin exocytosis, intact mitochondrial homeostasis and mild basal anti-inflammation; constitutively GTP-bound hyperactive ARF6 induces excessive mitochondrial fission, ferroptosis and overactive NF-κB signaling to aggravate β-cell failure and T2DM. Thus, optimal ARF6-directed therapies aim to restore physiological nucleotide cycling rather than fully suppress or permanently activate this GTPase. Accurately, ARF6 exhibits dual, tissue-specific metabolic functions and mediates adipose-pancreas interorgan lipotoxic crosstalk, forming a key translational obstacle for broad-spectrum ARF6 intervention. Moderate physiological ARF6 signaling in pancreatic β-cells promotes GSIS through the Cdc42/Rac1 cascade [5]; in contrast, excessive ARF6 activity in vascular endothelial cells impairs endothelium-mediated vasodilation and peripheral glucose delivery, driving systemic insulin resistance [20]. Meanwhile, sustained ARF6 hyperactivation in adipocytes triggers mitochondrial dysfunction and secretion of circulating lipotoxic and proinflammatory mediators—saturated fatty acids, ceramides, TNF-α, and IL-6 [69]. These circulating ligands bind β-cell GPR40 and TLR4 receptors, triggering transient compensatory β-cell proliferation followed by irreversible lipotoxic secretory failure. Notably, in vivo knockout evidence verifying this ligand–receptor signaling axis between adipose and pancreatic tissue is still lacking [70]. Collectively, these tissue-disparate ARF6 phenotypes and unvalidated interorgan signaling circuits necessitate tissue-targeted ARF6 modulators to separate therapeutic benefits from off-tissue toxic effects in metabolic disease treatment.

This study highlights the critical role of ARF6 in insulin secretion and metabolic disorders, providing new molecular insights into the pathogenesis of metabolic diseases, including T2DM and obesity. ARF6 participates in insulin secretion and lipid metabolism by regulating signaling pathways such as Cdc42 and Rac1, suggesting that it is a crucial pathological mediator and a potential therapeutic target. Further research into ARF6’s role in metabolic disorders could offer molecular mechanisms to enhance the efficacy of existing drugs, such as GLP-1RAs and metformin, and aid in developing novel treatments, particularly for refractory diabetes and obesity-related metabolic diseases. Although this study systematically evaluates the role of ARF6 in metabolic disorders through a combination of literature review, several limitations remain (Figure 1). However, existing ARF6 research yields contradictory outcomes determined by ARF activity magnitude, cell type and experimental models. Current evidence has prominent limitations: heavy reliance on rodent cell/HFD mouse data, insufficient human islet/adipose clinical specimens, and absent long-term compound safety assays. Key unresolved gaps cover interorgan adipocyte-β-cell signaling mediators and tissue-selective ARF modulator design. While ARF6 acts as a central metabolic hub for intervention, tissue-divergent effects and its narrow physiological activity window create major translational obstacles. Markedly, pan-ARF6 pathway blockade poses potential long-term safety risks, as basal ARF6 activity is indispensable for immune cell migration, endothelial repair and wound healing. Current short-term (8-week) high-fat diet mouse studies of SH-BC-893 demonstrate preserved macrophage chemotaxis and normal cutaneous wound closure, attributed to its selective inhibition of ceramide-triggered pathological ARF6 endocytic recycling rather than universal ARF6 physiological cycling. However, long-term chronic toxicity data (≥12 months continuous dosing) remain absent, representing a major translational safety gap. To resolve this limitation, next-generation ARF6 modulators should be engineered to selectively target stress-induced hyperactive ARF6 without disrupting basal homeostatic ARF6 function, combined with adipose-tropic targeted delivery to spare immune and epithelial tissue ARF6 signaling.

## 5. Future Directions and Conclusions

Future research should incorporate larger-scale clinical samples and multi-center studies to validate further the mechanisms and clinical relevance of ARF6 in metabolic disorders. Emerging analytical and experimental tools will deepen ARF6 research: single-cell transcriptomics and spatial omics map cell-type-specific ARF6 expression in metabolic organs; CRISPR editing generates tissue-specific ARF6 mutant models for functional verification; patient-derived pancreatic organoids mitigate limitations of rodent models; multi-omics profiling enables screening of ARF6-related circulating biomarkers for early metabolic disorder diagnosis. Long-term in vivo safety profiling of ARF6 modulators and the development of tissue-specific delivery platforms are urgently required to overcome the systemic risks of pan-ARF6 inhibition.

Currently, most ARF6 metabolic studies use mouse high-fat diet models and rodent beta cell lines. Islet structure and GSIS kinetics differ greatly between mice and humans, which limits translational value. Experiments on isolated human primary islets confirm the core ARF6-Cdc42/Rac1 insulin secretion pathway. However, we still lack large clinical human tissue data. No published work tracks how ARF6 activity changes as metabolic diseases worsen in patients. Future translational research should integrate multi-stage patient visceral adipose and pancreatic islet biobanks, human stem cell-derived pancreatic organoids and single-cell spatial omics to quantify ARF6 activity gradients from prediabetes to advanced T2DM, establishing clinical correlations between ARF6 signaling intensity and β-cell secretory reserve, insulin resistance severity and obesity grade.

## Data Availability

No new data were created or analyzed in this study. Data sharing is not applicable to this article.

## References

[1] Reiner D.J., Lundquist E.A. (2018). Small GTPases. WormBook.

[2] Fujibayashi K., Mima J. (2021). The Small GTPase Arf6 Functions as a Membrane Tether in a Chemically-Defined Reconstitution System. Front. Cell Dev. Biol..

[3] Møller L.L.V., Klip A., Sylow L. (2019). Rho GTPases-Emerging Regulators of Glucose Homeostasis and Metabolic Health. Cells.

[4] Veluthakal R., Thurmond D.C. (2021). Emerging Roles of Small GTPases in Islet β-Cell Function. Cells.

[5] Jayaram B., Syed I., Kyathanahalli C.N., Rhodes C.J., Kowluru A. (2011). Arf nucleotide binding site opener [ARNO] promotes sequential activation of Arf6, Cdc42 and Rac1 and insulin secretion in INS 832/13 β-cells and rat islets. Biochem. Pharmacol..

[6] Veluthakal R., Kaetzel D., Kowluru A. (2013). Nm23-H1 regulates glucose-stimulated insulin secretion in pancreatic β-cells via Arf6-Rac1 signaling axis. Cell. Physiol. Biochem..

[7] Zhao L., Ma J., Wang S., Xie Y. (2015). Relationship between β-cell function, metabolic control, and microvascular complications in type 2 diabetes mellitus. Diabetes Technol. Ther..

[8] Cho Y., Seo S.H., Seo D.H., Ahn S.H., Hong S., Huh B.W., Lee Y.H., Park S.W., Suh Y.J., Kim S.H. (2022). Differences in complication patterns in subgroups of type 2 diabetes according to insulin resistance and beta-cell function. Sci. Rep..

[9] Cheng Z., Almeida F.A. (2014). Mitochondrial alteration in type 2 diabetes and obesity: An epigenetic link. Cell Cycle.

[10] Armandi A., Rosso C., Caviglia G.P., Bugianesi E. (2021). Insulin Resistance across the Spectrum of Nonalcoholic Fatty Liver Disease. Metabolites.

[11] Afarideh M., Aryan Z., Ghajar A., Ganji M., Ghaemi F., Saadat M., Heidari B., Mechanick J.I., Esteghamati A. (2019). Association of non-alcoholic fatty liver disease with microvascular complications of type 2 diabetes. Prim. Care Diabetes.

[12] Schröder B., Kahl S., Roden M. (2021). Non-alcoholic fatty liver disease in type 2 diabetes—A specific entity?. Liver Int. Off. J. Int. Assoc. Study Liver.

[13] Dongiovanni P., Paolini E., Corsini A., Sirtori C.R., Ruscica M. (2021). Nonalcoholic fatty liver disease or metabolic dysfunction-associated fatty liver disease diagnoses and cardiovascular diseases: From epidemiology to drug approaches. Eur. J. Clin. Investig..

[14] Eslam M., George J. (2020). Genetic contributions to NAFLD: Leveraging shared genetics to uncover systems biology. Nat. Rev. Gastroenterol. Hepatol..

[15] Yeh K.H., Hsu L.A., Teng M.S., Wu S., Chou H.H., Ko Y.L. (2022). Pleiotropic Effects of Common and Rare GCKR Exonic Mutations on Cardiometabolic Traits. Genes.

[16] Nisar T., Arshad K., Abbas Z., Khan M.A., Safdar S., Shaikh R.S., Saeed A. (2023). Prevalence of GCKR rs1260326 Variant in Subjects with Obesity Associated NAFLD and T2DM: A Case-Control Study in South Punjab, Pakistan. J. Obes..

[17] Li L., Yu X.J., Gao L., Cheng L., Sun B., Wang G. (2022). Diabetic Ferroptosis and Pancreatic Cancer: Foe or Friend?. Antioxid. Redox Signal..

[18] Kusminski C.M., Ghaben A.L., Morley T.S., Samms R.J., Adams A.C., An Y., Johnson J.A., Joffin N., Onodera T., Crewe C. (2020). A Novel Model of Diabetic Complications: Adipocyte Mitochondrial Dysfunction Triggers Massive β-Cell Hyperplasia. Diabetes.

[19] Wang C.H., Wei Y.H. (2017). Role of mitochondrial dysfunction and dysregulation of Ca^2+^ homeostasis in the pathophysiology of insulin resistance and type 2 diabetes. J. Biomed. Sci..

[20] Islam M.T., Cai J., Allen S., Moreno D.G., Bloom S.I., Bramwell R.C., Mitton J., Horn A.G., Zhu W., Donato A.J. (2024). Endothelial-Specific Reduction in Arf6 Impairs Insulin-Stimulated Vasodilation and Skeletal Muscle Blood Flow Resulting in Systemic Insulin Resistance in Mice. Arterioscler. Thromb. Vasc. Biol..

[21] Ye H., Wang R., Wei J., Wang Y., Zhang X., Wang L. (2022). Bioinformatics Analysis Identifies Potential Ferroptosis Key Gene in Type 2 Diabetic Islet Dysfunction. Front. Endocrinol..

[22] Van Acker T., Tavernier J., Peelman F. (2019). The Small GTPase Arf6: An Overview of Its Mechanisms of Action and of Its Role in Host-Pathogen Interactions and Innate Immunity. Int. J. Mol. Sci..

[23] Chen K.J., Hsu J.W., Lee F.S. (2022). AMPK promotes Arf6 activation in a kinase-independent manner upon glucose starvation. J. Cell Sci..

[24] Grossmann A.H., Zhao H., Jenkins N., Zhu W., Richards J.R., Yoo J.H., Winter J.M., Rich B., Mleynek T.M., Li D.Y. (2019). The small GTPase ARF6 regulates protein trafficking to control cellular function during development and in disease. Small GTPases.

[25] Nikolatou K., Bryant D.M., Sandilands E. (2023). The ARF GTPase regulatory network in collective invasion and metastasis. Biochem. Soc. Trans..

[26] Uddin M.S., Kabir M.T., Jakaria M., Mamun A.A., Niaz K., Amran M.S., Barreto G.E., Ashraf G.M. (2019). Endothelial PPARγ Is Crucial for Averting Age-Related Vascular Dysfunction by Stalling Oxidative Stress and ROCK. Neurotox. Res..

[27] Walsh T.G., Li Y., Wersäll A., Poole A.W. (2019). Small GTPases in platelet membrane trafficking. Platelets.

[28] Sun D., Guo Y., Tang P., Li H., Chen L. (2023). Arf6 as a therapeutic target: Structure, mechanism, and inhibitors. Acta Pharm. Sin. B.

[29] Kowluru A. (2010). Small G proteins in islet beta-cell function. Endocr. Rev..

[30] Stalin A., Hesham A.E., Mishra A., Zou Q., Ignacimuthu S. (2024). Editorial: Herbal medical products for metabolic diseases—New integrated pharmacological approaches. Front. Pharmacol..

[31] Wang S., Lu Y., Chi T., Zhang Y., Zhao Y., Guo H., Feng L. (2023). Identification of ferroptosis-related genes in type 2 diabetes mellitus based on machine learning. Immun. Inflamm. Dis..

[32] Deepa Maheshvare M., Raha S., König M., Pal D. (2023). A pathway model of glucose-stimulated insulin secretion in the pancreatic β-cell. Front. Endocrinol..

[33] Piona C., Mozzillo E., Tosco A., Zusi C., Emiliani F., Volpi S., Di Candia F., Raia V., Boselli M.L., Trombetta M. (2025). A Longitudinal Study of Glucose Tolerance in Cystic Fibrosis: The Central Role of Beta Cell Functional Mass. J. Clin. Endocrinol. Metab..

[34] Naude C.E., Brand A., Schoonees A., Nguyen K.A., Chaplin M., Volmink J. (2022). Low-carbohydrate versus balanced-carbohydrate diets for reducing weight and cardiovascular risk. Cochrane Database Syst. Rev..

[35] Khin P.P., Lee J.H., Jun H.-S. (2023). Pancreatic Beta-cell Dysfunction in Type 2 Diabetes. Eur. J. Inflamm..

[36] Guo R., Jiang J., Jing Z., Chen Y., Shi Z., Deng B. (2018). Cysteinyl leukotriene receptor 1 regulates glucose-stimulated insulin secretion (GSIS). Cell. Signal..

[37] Sidarala V., Kowluru A. (2017). Exposure to chronic hyperglycemic conditions results in Ras-related C3 botulinum toxin substrate 1 (Rac1)-mediated activation of p53 and ATM kinase in pancreatic β-cells. Apoptosis Int. J. Program. Cell Death.

[38] Kowluru A. (2021). Roles of GTP and Rho GTPases in pancreatic islet beta cell function and dysfunction. Small GTPases.

[39] Finicle B.T., Eckenstein K.H., Revenko A.S., Anderson B.A., Wan W.B., McCracken A.N., Gil D., Fruman D.A., Hanessian S., Seth P.P. (2023). Simultaneous inhibition of endocytic recycling and lysosomal fusion sensitizes cells and tissues to oligonucleotide therapeutics. Nucleic Acids Res..

[40] Jayashankar V., Selwan E., Hancock S.E., Verlande A., Goodson M.O., Eckenstein K.H., Milinkeviciute G., Hoover B.M., Chen B., Fleischman A.G. (2021). Drug-like sphingolipid SH-BC-893 opposes ceramide-induced mitochondrial fission and corrects diet-induced obesity. EMBO Mol. Med..

[41] Muley C., Bartelt A. (2021). Fuse your mitochondria, lose appetite: An anorexic, anti-obesity sphingolipid. EMBO Mol. Med..

[42] Ruze R., Liu T., Zou X., Song J., Chen Y., Xu R., Yin X., Xu Q. (2023). Obesity and type 2 diabetes mellitus: Connections in epidemiology, pathogenesis, and treatments. Front. Endocrinol..

[43] Thamilselvan V., Kowluru A. (2021). Paradoxical regulation of glucose-induced Rac1 activation and insulin secretion by RhoGDIβ in pancreatic β-cells. Small GTPases.

[44] Yang Y., Cai Z., Pan Z., Liu F., Li D., Ji Y., Zhong J., Luo H., Hu S., Song L. (2021). Rheb1 promotes glucose-stimulated insulin secretion in human and mouse β-cells by upregulating GLUT expression. Metab. Clin. Exp..

[45] Lipták N., Bősze Z., Hiripi L. (2019). GFP transgenic animals in biomedical research: A review of potential disadvantages. Physiol. Res..

[46] Lawrence J.T., Birnbaum M.J. (2003). ADP-ribosylation factor 6 regulates insulin secretion through plasma membrane phosphatidylinositol 4,5-bisphosphate. Proc. Natl. Acad. Sci. USA.

[47] Santy L.C., Casanova J.E. (2001). Activation of ARF6 by ARNO stimulates epithelial cell migration through downstream activation of both Rac1 and phospholipase D. J. Cell Biol..

[48] Yamaoka M., Ando T., Terabayashi T., Okamoto M., Takei M., Nishioka T., Kaibuchi K., Matsunaga K., Ishizaki R., Izumi T. (2016). PI3K regulates endocytosis after insulin secretion by mediating signaling crosstalk between Arf6 and Rab27a. J. Cell Sci..

[49] López-Gambero A.J., Martínez F., Salazar K., Cifuentes M., Nualart F. (2019). Brain Glucose-Sensing Mechanism and Energy Homeostasis. Mol. Neurobiol..

[50] Deng S., Yang L., Ma K., Bian W. (2021). Astragalus polysaccharide improve the proliferation and insulin secretion of mouse pancreatic β cells induced by high glucose and palmitic acid partially through promoting miR-136-5p and miR-149-5p expression. Bioengineered.

[51] Sumithran P., Ard J. (2025). The promise and hope of GLP-1 receptor agonists. Lancet Diabetes Endocrinol..

[52] Hoeg-Jensen T. (2021). Review: Glucose-sensitive insulin. Mol. Metab..

[53] Li Y., Yang B., Shi C., Tan Y., Ren L., Mokrani A., Li Q., Liu S. (2023). Integrated analysis of mRNAs and lncRNAs reveals candidate marker genes and potential hub lncRNAs associated with growth regulation of the Pacific Oyster, *Crassostrea gigas*. BMC Genom..

[54] Nguyen T.P., Jacobs P.G., Castle J.R., Wilson L.M., Kuehl K., Branigan D., Gabo V., Guillot F., Riddell M.C., Haidar A. (2021). Separating insulin-mediated and non-insulin-mediated glucose uptake during and after aerobic exercise in type 1 diabetes. Am. J. Physiol. Endocrinol. Metab..

[55] Elenjickal E.J., Valson A.T., Varughese S., Vincent L., Fernando E., Natarajan G. (2024). Editorial: Novel pathophysiologic mechanisms and reno-protective pharmacotherapies in diabetic kidney disease. Front. Pharmacol..

[56] Ladenheim E.E. (2015). Liraglutide and obesity: A review of the data so far. Drug Des. Dev. Ther..

[57] Hsu S.K., Cheng K.C., Mgbeahuruike M.O., Lin Y.H., Wu C.Y., Wang H.D., Yen C.H., Chiu C.C., Sheu S.J. (2021). New Insight into the Effects of Metformin on Diabetic Retinopathy, Aging and Cancer: Nonapoptotic Cell Death, Immunosuppression, and Effects beyond the AMPK Pathway. Int. J. Mol. Sci..

[58] Dhameja M., Gupta P. (2019). Synthetic heterocyclic candidates as promising α-glucosidase inhibitors: An overview. Eur. J. Med. Chem..

[59] Ye Y., Barghouth M., Dou H., Luan C., Wang Y., Karagiannopoulos A., Jiang X., Krus U., Fex M., Zhang Q. (2022). A critical role of the mechanosensor PIEZO1 in glucose-induced insulin secretion in pancreatic β-cells. Nat. Commun..

[60] Staels B., Handelsman Y., Fonseca V. (2010). Bile acid sequestrants for lipid and glucose control. Curr. Diabetes Rep..

[61] Cho D.I., Zheng M., Min C., Kwon K.J., Shin C.Y., Choi H.K., Kim K.M. (2013). ARF6 and GASP-1 are post-endocytic sorting proteins selectively involved in the intracellular trafficking of dopamine D_2_ receptors mediated by GRK and PKC in transfected cells. Br. J. Pharmacol..

[62] Castanet A.S., Nafie M.S., Said S.A., Arafa R.K. (2023). Discovery of PIM-1 kinase inhibitors based on the 2,5-disubstituted 1,3,4-oxadiazole scaffold against prostate cancer: Design, synthesis, in vitro and in vivo cytotoxicity investigation. Eur. J. Med. Chem..

[63] Ard J., Fitch A., Fruh S., Herman L. (2021). Weight Loss and Maintenance Related to the Mechanism of Action of Glucagon-Like Peptide 1 Receptor Agonists. Adv. Ther..

[64] Cheng X., Sun P., Yan J., Zhai X., Liu W., Li D., Hong J., Cao Z., Jiang B. (2026). Genome-wide identification and expression analysis of the ARF gene family in wax gourd (*Benincasa hispida*). BMC Plant Biol..

[65] Benjamin M.A., McKay D.M., Yang P.C., Cameron H., Perdue M.H. (2000). Glucagon-like peptide-2 enhances intestinal epithelial barrier function of both transcellular and paracellular pathways in the mouse. Gut.

[66] Castañeda S., Remuzgo-Martínez S., López-Mejías R., Genre F., Calvo-Alén J., Llorente I., Aurrecoechea E., Ortiz A.M., Triguero A., Blanco R. (2019). Rapid beneficial effect of the IL-6 receptor blockade on insulin resistance and insulin sensitivity in non-diabetic patients with rheumatoid arthritis. Clin. Exp. Rheumatol..

[67] Fiola-Masson É., Campbell S., Laplante V., Lejri R., Kanaho Y., Gauchat J.F., Servant M.J., Claing A. (2026). ARF6 controls VSMC phenotypic switching upon lipid stimulation to promote inflammatory signaling contributing to the progression of atherosclerosis. J. Biol. Chem..

[68] Hafner M., Schmitz A., Grüne I., Srivatsan S.G., Paul B., Kolanus W., Quast T., Kremmer E., Bauer I., Famulok M. (2006). Inhibition of cytohesins by SecinH3 leads to hepatic insulin resistance. Nature.

[69] Liu Y., Zhou D., Abumrad N.A., Su X. (2010). ADP-ribosylation factor 6 modulates adrenergic stimulated lipolysis in adipocytes. Am. J. Physiol. Cell Physiol..

[70] Li J., Chen L., Zhang Y., Zhang W.J., Xu W., Qin Y., Xu J., Zou D. (2013). TLR4 is required for the obesity-induced pancreatic beta cell dysfunction. Acta Biochim. Biophys. Sin..

